# ZMYM2 inhibits NANOG-mediated reprogramming

**DOI:** 10.12688/wellcomeopenres.15250.1

**Published:** 2019-06-06

**Authors:** Moyra Lawrence, Thorold W. Theunissen, Patrick Lombard, David J. Adams, José C. R. Silva

**Affiliations:** 1 Wellcome-MRC Cambridge Stem Cell Institute, University of Cambridge, Cambridge, Cambridgeshire, CB2 1QR, UK; 2 Department of Biochemistry, University of Cambridge, Cambridge, CB2 1QR, UK; 3 Department of Developmental Biology and Center of Regenerative Medicine, Washington University School of Medicine, St. Louis, MO, 63110, USA; 4 Experimental Cancer Genetics, Wellcome Sanger Institute, Cambridge, CB10 1SA, UK

**Keywords:** Nanog, reprogramming, Zinc finger protein, pluripotency, differentiation

## Abstract

**Background:** NANOG is a homeodomain-containing transcription factor which forms one of the hubs in the pluripotency network and plays a key role in the reprogramming of somatic cells and epiblast stem cells to naïve pluripotency.  Studies have found that NANOG has many interacting partners and some of these were shown to play a role in its ability to mediate reprogramming. In this study, we set out to analyse the effect of NANOG interactors on the reprogramming process.

**Methods:** Epiblast stem cells and somatic cells were reprogrammed to naïve pluripotency using MEK/ERK inhibitor PD0325901, GSK3β inhibitor CHIR99021 and Leukaemia Inhibitory Factor (together termed 2i Plus LIF). *Zmym2* was knocked out using the CRISPR/Cas9 system or overexpressed using the PiggyBac system. Reprogramming was quantified after ZMYM2 deletion or overexpression, in diverse reprogramming systems. In addition, embryonic stem cell self renewal was quantified in differentiation assays after ZMYM2 removal or overexpression.

**Results:** In this work, we identified ZMYM2/ZFP198, which physically associates with NANOG as a key negative regulator of NANOG-mediated reprogramming of both epiblast stem cells and somatic cells. In addition, ZMYM2 impairs the self renewal of embryonic stem cells and its overexpression promotes differentiation.

**Conclusions:** We propose that ZMYM2 curtails NANOG’s actions during the reprogramming of both somatic cells and epiblast stem cells and impedes embryonic stem cell self renewal, promoting differentiation.

## Introduction

Reprogramming is the process whereby a somatic cell is reverted back to a pluripotent state. Pluripotent cells possess the ability both to self-renew and to differentiate into cells from any of the three germ layers of the adult organism. Reprogramming can be carried out by overexpressing only four factors in somatic cells: *Oct4*, *Klf4*, *Sox2* and *cMyc* ^1^. Together, these factors reset the transcriptional and epigenetic state of the cell to those of a pluripotent cell. Much work has been carried out on factors which can execute or promote this transition. These include many members of the pluripotency-associated transcription factor network ^2– 7^. *Nanog* is a homeodomain-containing transcription factor which constitutes one of these key factors.

*Nanog* was first discovered for its ability to promote embryonic stem cell (ESC) self-renewal in the absence of LIF and for its association with the pluripotent state as opposed to somatic identities ^8, 9^. *Nanog* is also essential for the establishment of the pluripotent naïve epiblast ^10^. Thus, *Nanog* plays a central role in the promotion of the pluripotent state, both *in vitro* and *in vivo*.

As a key hub of the pluripotency network, studies have been carried out aiming at understanding *Nanog*’s mode of action. One approach was to define its interactome, which led to the identification of multiple interactors ^3, 11, 12^. Some of these are chromatin modifiers that were shown to augment the ability of *Nanog* to mediate reprogramming. These include the NuRD complex ^13^ and the TET family proteins ^11^. Importantly, we still do not know if most of the identified interactors play a role, either positive or negative, in the mechanism of action of *Nanog*. In order to address this, we set out to analyse the effect of additional NANOG interactors on *Nanog*-mediated reprogramming. This work enabled us to identify ZMYM2/ZFP198, which physically associates with NANOG ^3, 11, 12, 14^, as a key protein impairing *Nanog*’s activity in both reprogramming and the self-renewal of naïve pluripotent stem cells.

## Methods

### Cell culture

Mouse ESCs, iPS cells and pre-iPS cells were cultured in Glasgow Minimum Essential Medium (GMEM; Sigma, G5154) containing 10% foetal calf serum (FCS; Life Technologies, 10091-148), 1x non-essential amino acids solution (NEAA; PAA, M11-003), 1 mM sodium pyruvate (PAA, S11-003), 0.1 mM 2-mercaptoethanol (Invitrogen 31350-010), 2mM L-glutamine (Invitrogen, 25030024), 1x Pen/Strep (PAA, P11010) and 20 ng/mL LIF (Department of Biochemistry, University of Cambridge). This medium will hereafter be referred to as Serum Plus LIF. These cells were grown on plastic dishes (Iwaki/Corning, 10578911) which had treated with 0.1% gelatin for 10 min. Fibroblasts were cultured in GMEM (Sigma, G5154) containing 10% FCS (Life Technologies, 10091-148) on gelatin-coated dishes.

N2B27-containing medium was made up as follows: 50% neurobasal (Life Technologies, 21103-049), 50% Dulbecco's Modified Eagle Medium (DMEM)/F12 (Life Technologies, 11330-057), 1X N2 (WT/MRC SCI, University of Cambridge), 1X B27 (Life Technologies, 17504-044), 2mM L-glutamine (Invitrogen, 25030024), 1X Pen/Strep, 0.1 mM 2-mercaptoethanol (Invitrogen 31350-010).

Epiblast stem cells (EpiSCs) were cultured in N2B27-containing medium supplemented with 12.5 ng/mL FGF2 (WT/MRC SCI, University of Cambridge) and 20 ng/mL Activin A (WT/MRC SCI, University of Cambridge). They were grown on dishes which had been coated with 10 μg/mL Human recombinant fibronectin (Millipore FC010) in PBS for 30 min at room temperature.

Neural stem cells (NSCs) were cultured in DMEM/F12 (Life Technologies, 11330-057) containing 27.4mM glucose, 1x NEAA (PAA, M11-003), 1X Pen/Strep (PAA, P11010), 4mM HEPES (Life Technologies, 15630-049), 0.011% Bovine serum albumin, 1X N2 (WT/MRC SCI, University of Cambridge), 1X B27 (Life Technologies, 17504-044), 0.1 mM 2-mercaptoethanol (Invitrogen 31350-010), 10 ng/mL of epidermal growth factor (EGF; Peprotech, 315-09) and 20 ng/mL fibroblast growth factor 2 (FGF2; WT/MRC SCI, University of Cambridge). They were cultured on plastic dishes which had been coated for at least 3 h with 10 μg/mL laminin (Sigma, L2020) in PBS and washed once in PBS. 

### Cell lines

Oct4 reporter EpiSCs and NSCs were used as previously detailed and contained an Oct4-GFP-IRES-puro reporter transgene in which enhanced green fluorescent protein (eGFP) is expressed under the control of Oct4 (Pou5f1) regulatory elements ^15, 16^. Nanog-GFP-IRES-puro reporter NSCs were also used as previously generated and these contained GFP inserted heterozygously into the AUG start codon of one endogenous *Nanog* allele ^11, 17^. Nanog ^-/-^ pre-iPSCs had been previously generated in the lab by the retroviral transduction of Nanog ^-/-^ NSCs isolated from E12.5 forebrain with *Oct4*, *Klf4* and *cMyc* ^10^ E14tg2a ESCs were used for all self-renewal assays ^18^.

### siRNA transfection

FlexiTube siRNA solutions (Qiagen) were used to knock down expression of the following genes: *Zmym2* (GS76007), *Zfp281* (GS226442) and *Nr0b1* (GS11614). All Star negative control siRNA was also used (1027281). Transfection was carried out with Lipofectamaine RNAi Max (Life Technologies, 13778030). Medium was changed to medium containing MEK/ERK inhibitor PD0325901, GSK3β inhibitor CHIR99021 (WT/MRC SCI, University of Cambridge) and Leukaemia Inhibitory Factor (Department of Biochemistry, University of Cambridge) (together termed 2i Plus LIF) ^19^ with Penicillin/Streptomycin 24 h after transfection and the cells were allowed to reprogram for 12 days. Green colonies, resulting from the expression of a Oct4-GFP reporter ^11, 13, 20^, were monitored using a Leica epifluorescent DMI4000 microscope at 488nm as a readout of reprogramming efficiency.

### Measurement of pluripotency-associated gene expression by qPCR

Total RNA was extracted from cells using an RNeasy mini kit (Qiagen, 74106), with DNAse treatment (Qiagen, 79254). cDNA synthesis was performed using the Superscript III kit (Life Technologies, 11752-250) in accordance with the manufacturer’s protocol. RT-qPCR was carried out in microAmp qPCR plates (Life Technologies, 434690) on a StepOne Plus Real-Time PCR machine (Applied Biosystems) using TaqMan Fast Universal MasterMix (Applied Biosystems, 4352042) and expression levels were calculated by ΔCt to *Gapdh*. Mean expression levels were determined by averaging triplicate wells. TaqMan amplification was performed as follows: 2 min at 50 °C, 20 sec at 95 °C, (1 sec at 95 °C, 20 sec at 60 °C) x 40. Probes used are presented in Table 1.

**Table 1.** Probes used for qPCR.

Probe	Applied Biosystems ID
*Klf4*	Mm00516104_m1
*Klf2*	Mm01244979_g1
*Rex1*	Mm03053975_g1
*Nr0b1*	Mm00431729_m1
*Oct4*	Mm00658129_gH
*Zmym2*	Mm00813221_m1
*Esrrb*	Mm00442411_m1
*Nanog*	Mm02384862_g1
*Gapdh*	Mm99999915_g1

### Reprogramming neural stem cells and mouse embryonic fibroblasts

Retroviral reprogramming vectors (pMXs- *Oct4* (13366), pMXs- *Klf4* (13370), pMXs- *Sox2* (13367) and pMXs- *cMyc* (13375)) were obtained from the Addgene repository. PLAT-E cells were transfected with these using FuGene (Promega E2311). The medium containing retroviral particles was collected from the PLAT-E cells and filtered through a 0.45 μm filter. Neural stem cells (NSCs) were transduced with retroviral (r) *Oct4*, *cMyc* and *Klf4* whereas MEFs were transduced with these and *rSox2*. 4μg/ml polybrene (Sigma Aldrich, TR-1003) was added for transduction.

24 h after transduction, the virus-containing medium was aspirated from the NSCs or MEFs and replaced with the cells’ respective media. Four days after transduction, the medium was replaced with Serum-containing medium supplemented with Leukaemia Inhibitory Factor ^21^ (Serum Plus LIF). The cells slowly became more proliferative and acquired pre-iPS cell-like morphology. If the pre-iPS were being reprogrammed in the same well, the medium was switched to medium containing MEK/ERK inhibitor PD0325901, GSK3β inhibitor CHIR99021 (WT/MRC SCI, University of Cambridge) and Leukaemia Inhibitory Factor (Department of Biochemistry, University of Cambridge) (together termed 2i Plus LIF) ^19^ 4 days after the application of Serum Plus LIF (8 days after retroviral transduction).

Cells were then stably transfected with PiggyBac (PB) *Nanog* transgenes, selected and subjected to transient transfection with siRNA before reprogramming in 2i Plus LIF. Oct4- or Nanog-GFP ^+^ colonies were counted 12 days later (see cell line section for details).

### Zmym2 overexpression

Overexpression vectors were generated using Gateway cloning (Invitrogen) and PiggyBac vectors. Cells were transfected using Lipofectamine 2000 (ThermoFisher Scientific 11668019) and selected for 14 days with either hygromycin or blasticidin (WT/MRC SCI, University of Cambridge).

### CRISPR/Cas9 generation of Zmym2 ^-/-^ EpiSC and NSC cells

A double-stranded break was induced 108 amino acids after the start codon of *Zmym2*, inducing frameshift mutations in both alleles. Pools of clones were screened by T7 assay ^22^, which involves the annealing of PCR products from the edited locus to PCR products from the WT locus. These double stranded fragments are then digested with T7 endonuclease, which cuts the imperfectly annealed strands. These cut products can then be visualised using agarose gel electrophoresis. Single transfected cells were then sorted and analysed for ZMYM2 knockout by Western Blotting (see Table 2 for antibodies used). Clones were selected which had no intact *Zmym2* alleles and were stably transfected with *Nanog* or *Zmym2* transgenes or both, or their corresponding empty vector transgenes.

**Table 2.** Antibodies used for western blots.

Target	Species	Clonality	Concentration	Dilution	Cat no	Supplier
alpha tubulin	mouse	Mono	1 mg/mL	1:5000	ab7291	Abcam
NANOG	rat	Mono	500 μg/mL	1:100	eBio MLC51	eBiosciences
OCT4 C10	mouse	Mono	200 μg/mL	1:500	sc-5279	Santa Cruz
ZMYM2	rabbit	Poly	400 μg/mL	1:440	ab30783	Abcam

### Antibodies

***Self-renewal assay for ESCs.*** *Zmym2* was stably overexpressed in ESCs or knocked out by CRISPR/Cas9 as detailed above. These cells were plated alongside Empty Vector (EV) controls in Serum-containing medium with or without LIF for 6 days. Alkaline-phosphatase staining (Sigma, 86R-1KT) was carried out and colonies were scored by both morphology and alkaline phosphatase staining.

### Transcriptome analysis

mRNA was extracted with a RNeasy kit (Qiagen, 74106), with DNAse treatment (Qiagen, 79254). It was quantified using Agilent Bioanalyzer Nano Chips (Agilent Technologies). Depletion of ribosomal RNA was performed on 2-5 μg of total RNA using the Ribo-Zero rRNA Removal Kit (Illumina) and libraries were produced from 10-100ng of ribosomal-depleted RNA using NextFlex Rapid Directional RNA-seq Kit (5138-07; Bioo Scientific), a Biorad C1000 thermocycler, and standard Illumina primers. Cycling conditions were as follows: 30 min at 37°C, 2 min at 98°C, (30 sec at 98°C, 30 sec at 65°C, 60 sec at 72°C) x 12, 4 min at 72°C. Libraries were pooled in equimolar quantities and sequenced on the HiSeq4000 platform (Illumina), using V4 chemistry.

RNA-seq reads were adaptor-trimmed with TrimGalore (version 0.3.7) and mapped to the mouse reference genome (GRCm38/mm10) with TopHat2 (version 2.2.3). Strand-specific read counts were obtained with featureCounts (version 1.4.5). Transcript counts were normalised, and the statistical significance of differential expression between samples was assessed using the R Bioconductor DESeq2 (version 1.4.5) package. Transcript counts normalized by DESeq2 size factors were subsequently normalized by their length.

### Blastocyst injection and animal husbandry

Chimeras were generated from mouse strain 129 (agouti coat color) iPSCs by standard microinjection methodology at the Wellcome Trust/MRC Cambridge Stem Cell Institute. Briefly, host blastocysts of strain C57BL/6 (black coat colour) were injected at E4.5, followed by gestation in pseudo-pregnant recipient females ^23^. These females were 6–10 weeks old and 25–30g. The resulting chimeras were then bred with WT mice and the pups analysed by coat colour for contribution of the iPS-derived cells. The use of animals in this project was approved by the Animal Welfare and Ethical Review Body for the University of Cambridge (Procedure Project Licenses P76777883 and 80/2597). Mice were housed in individual ventilated cages with up to 5 animals per cage. Stud males were individually caged and females were housed in groups, with wood chips and mouse bedding plugs on the cage floor. The mouse facility was a barrier facility with 10 hours darkness and 14 hours light per day. The temperature was maintained at 22 °C. Food and water were provided *ad libitum*. Cages contained environmental enrichment for the mice, including wooden blocks and perspex houses. All animals were checked on a daily basis by trained animal house staff, but there are no welfare issues expected from the embryo transfer procedure, which is performed routinely by the dedicated transgenic facility manager. Every effort was made to reduce the numbers of animals used and the stress or discomfort caused to animals in this study. The final assay result is coat colour of the pups, and did not involve any invasive or stressful procedures. Further details regarding the mice used are presented in Table 3.

**Table 3.** Details of mice used for chimera contribution assay. Zmym2 KD or OE iPSCs were injected into E4.5 C57Bl6 host blastocysts. The resulting embryos were implanted into pseudopregnant females and the pups analysed for iPS contribution by coat colour. M:male, F:female, GLT: germline transmission.

													Contribution			
Date	Clone name	No. of Females	Strain	No. of embryos	No. injected	No. transferred	No. recipients	No. pregnant	No. born	No. of chimeras	M	F	high	medium	Low	GLT
13/05/2013	Zmym2 KD	12	C57Bl6	74	70	70	5x25	2	17	5	3	2	0	5	0	Yes
14/06/2013	Zmym2 OE	9	C57Bl6	21	20	20	2x25	1	2	0	0	0	0	0	0	N/A

## Results

### *Zmym2* impairs *Nanog* -mediated reprogramming in EpiSCs

**Figure 1.**
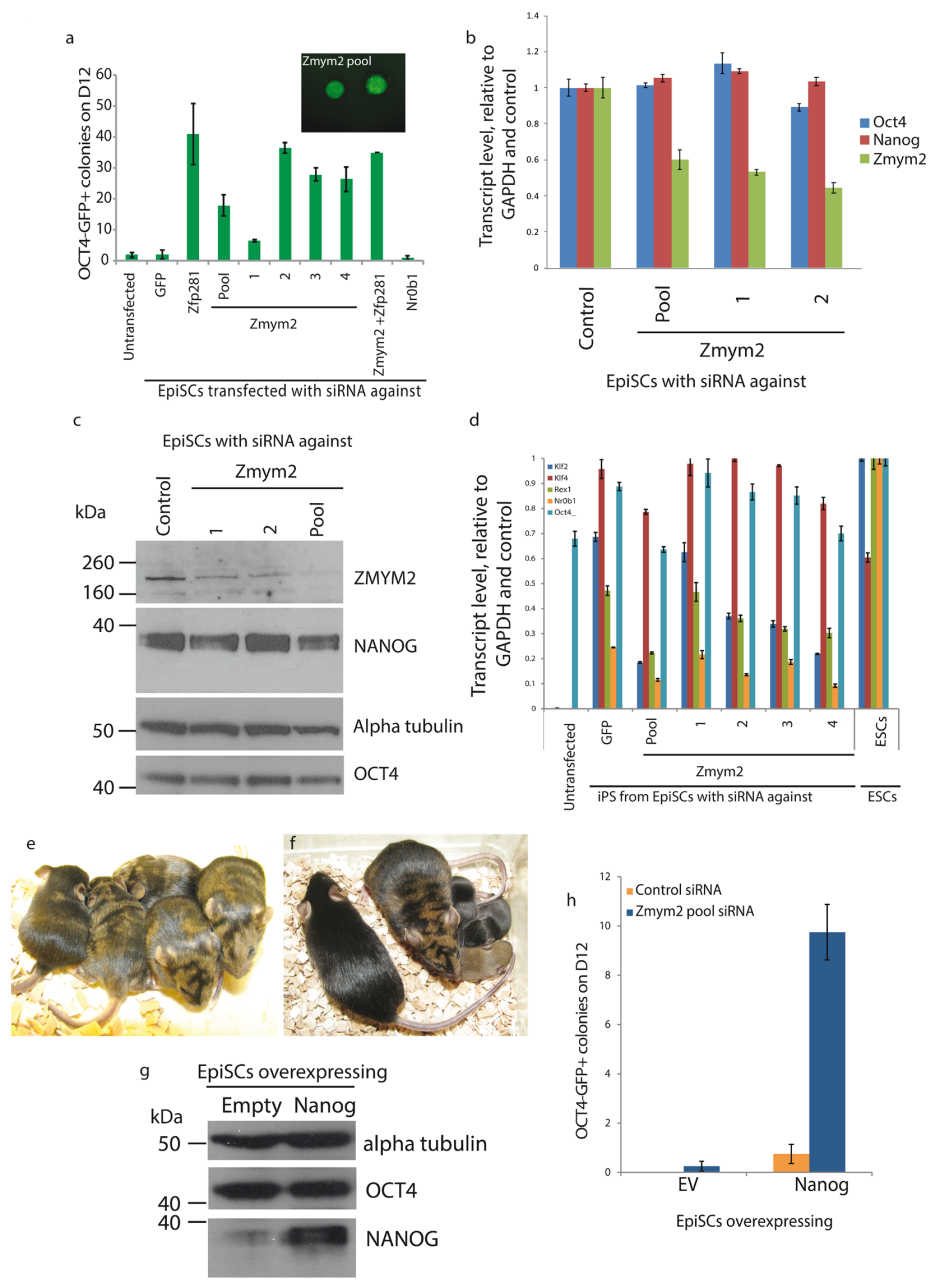
*Zmym2* is a repressor of *Nanog*-mediated epiblast stem cell (EpiSC) reprogramming. **a**, 2000 EpiSCs were transiently transfected with siRNA against the indicated targets and reprogrammed by *Nanog* overexpression in 2i Plus LIF. Oct4-GFP ^+^ colonies were scored on day 12. Data represent the mean number of Oct4-GFP ^+^ colonies from two replicates +/- SEM. Example colonies are shown in the inset panel. **b**, RT-qPCR analysis of EpiSC lines 48h after *Zmym2*KD. Data are the mean normalized expression level from 3 technical replicates +/- SD. **c**, Western Blot of EpiSC lines 48h after *Zmym2*KD with alpha tubulin shown as a loading control. **d**, Gene expression analysis of the parent EpiSCs and resulting iPSCs by qPCR. Data are the mean normalised expression levels from 3 technical replicates +/- SD. **e**, iPSCs which emerged from *Zmym2*KD EpiSCs were injected into C57Bl6 blastocysts to generate chimeras, which can be seen from their coat colour (brown fur conferred by iPSC contribution). **f**, Germline transmission of the iPSCs (brown pup from iPSCs shown with its chimera mother and black father). **g**, Western Blot of lines of empty vector (EV) and NANOG-overexpressing (Nanog) EpiSCs with alpha tubulin shown as a loading control. **h**, GFP ^+^ colony count of EV- and *Nanog*-overexpressing EpiSCs when reprogrammed in the presence of siRNA against *Zmym2* or control siRNA. Data represent the mean +/- SEM of two independent experiments.

In this study, we aimed to characterise potential regulators of *Nanog*’s activity during reprogramming. We compared NANOG interactomes ^3, 11, 12^ and selected ZMYM2 and NR0B1 as candidates of interest due to these being high confidence interactors. ZFP281 was selected as a control, as knocking it down had been previously demonstrated to increase *Nanog*-mediated reprogramming efficiency ^24^. In order to address whether these factors impact *Nanog*-induced reprogramming, *Nanog*-overexpressing EpiSCs, which reprogram at low efficiency ^10, 20^, were transiently transfected with siRNA against the target genes of interest ( Figure 1A). The medium was then swapped to medium containing the MEK/ERK inhibitor PD0325901 and the GSK3β inhibitor CHIR99021 ^19^. This medium will hereafter be referred to as (2i) plus LIF medium (2i Plus LIF). This medium promotes reprogramming ^19^. These cells express enhanced green fluorescent protein (eGFP), under the control of Oct4 (Pou5f1) regulatory elements, making the cells GFP ^+^ when fully reprogrammed to naïve pluripotency ^15, 16^. As a readout of reprogramming efficiency, Oct4-GFP ^+^ colonies were counted 12 days after the application of 2i Plus LIF. 

*Nr0b1* knockdown (KD) did not alter reprogramming efficiency. *Zfp281*KD increased *Nanog* mediated reprogramming efficiency, consistent with a previous report ^24^. Interestingly, reprogramming efficiency was robustly increased by *Zmym2*KD ( Figure 1A). Zmym2 transcript and protein levels were reduced by all four siRNAs by qPCR and by Western blot ( Figure 1B and 1C respectively), 48h after transfection. This contrasts with the action of many other NANOG interactors as activators of reprogramming ^11, 13^ and suggests that *Zmym2* impedes *Nanog*-mediated reprogramming. The iPSCs generated after *Zmym2*KD were characterised and had gene expression profiles consistent with the acquisition of naive pluripotency ( Figure 1D) and upon injection into C57Bl6 mouse host blastocysts, chimerae were produced ( Figure 1E). *Zmym2*KD iPSCs also exhibited germline competence ( Figure 1F). This indicates faithful iPSC reprogramming following *Zmym2*KD.

Given that these EpiSCs overexpressed *Nanog* to promote reprogramming, we then investigated whether *Zmym2*KD is sufficient to reprogram EpiSCs in the absence of any transgenic reprogramming factors. In order to address this, EpiSCs overexpressing *Nanog* or a corresponding empty vector (EV) transgene ( Figure 1G) were transfected with siRNA against *Zmym2* and transferred to reprogramming conditions. *Zmym2KD* had a pronounced positive effect on *Nanog*-induced reprogramming but a minimal effect on the reprogramming of EV EpiSCs ( Figure 1H). Therefore, *Zmym2*KD relies on the exogenous expression of *Nanog* in order to robustly enhance reprogramming.

### *Zmym2* impairs *Nanog* -mediated somatic cell reprogramming

**Figure 2.**
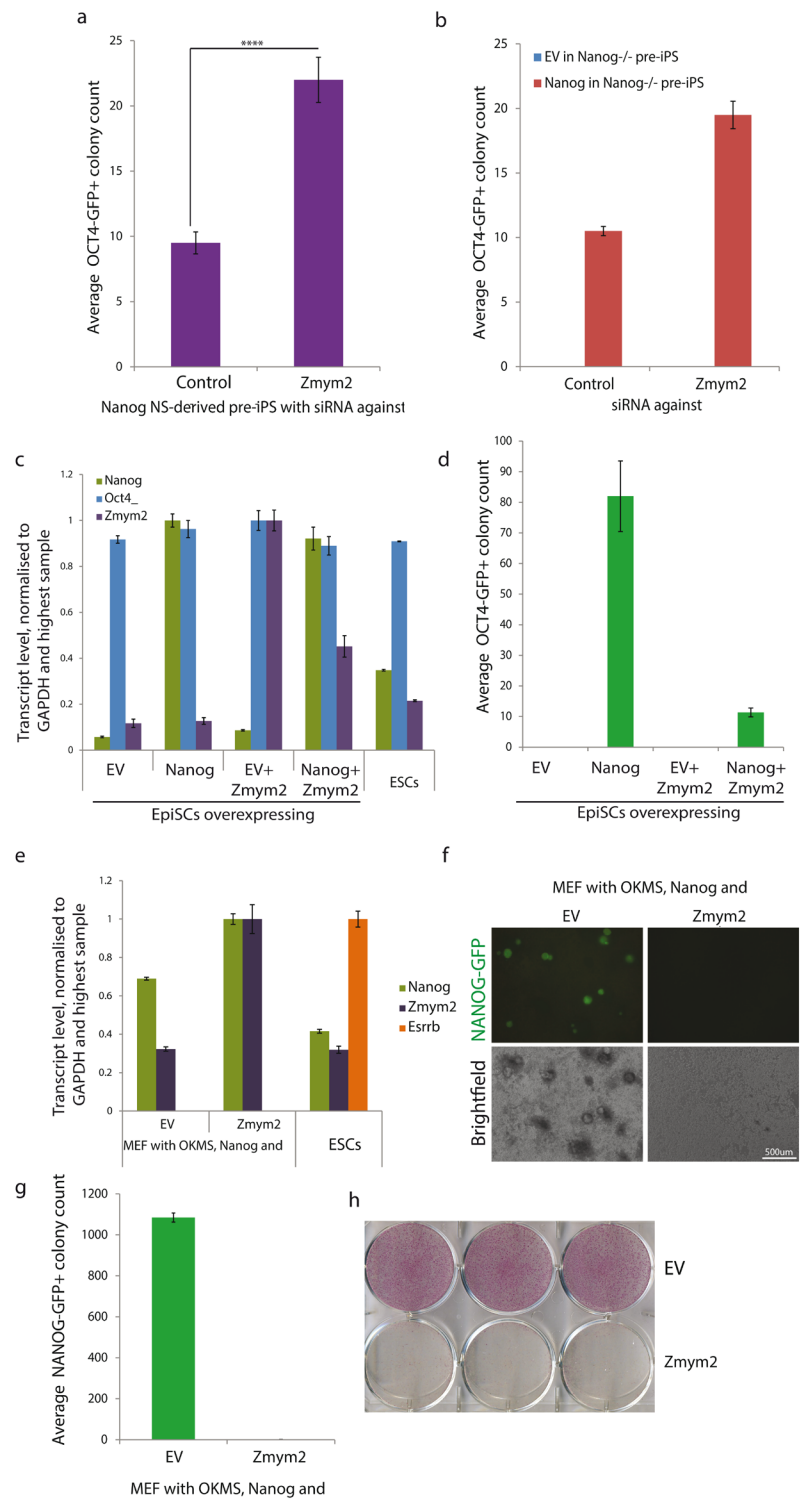
*Zmym2* inhibits somatic cell reprogramming in a *Nanog*-dependent manner. **a**, Neural stem cells (NSCs) were reprogrammed with retroviral *Oct4, Klf4 and cMyc* and with constitutive transgenic *Nanog* expression in 2i Plus LIF in the presence of *Zmym2* or control siRNA. Colony count per 75,000 plates NSCs. Average of three independent experiments. **** p<0.0005 by Student’s T-test. **b**, Average GFP ^+^ colony count per 10,000 *Nanog* ^-/-^ pre-iPSCs reprogrammed in the presence of *Zmym2* or control KD, two replicates +/-SEM **c**, Gene expression analysis of EpiSC lines stably overexpressing *Nanog*, *Zmym2,* or both by RT-qPCR. **d**, Average GFP ^+^ colony count on D12 after 2i Plus LIF application per 25,000 plated EpiSCs, three replicates. **e**, Gene expression analysis by qPCR of MEF-derived pre-iPSCs stably overexpressing *Nanog*+EV or *Nanog*+ *Zmym2*, with ESC control. **f**, Fluorescence and brightfield images of Oct4-GFP ^+^ colonies on D12 **g**, Average GFP ^+^ colony count on D12 per 50,000 plates pre-iPSCs, three replicates. **h**, Alkaline phosphatase staining on D12. Reprogramming counts are shown as mean +/-SEM. qPCR quantifications are shown as mean of three technical replicates +/- SD, normalised to GAPDH transcript levels.

All experiments described so far had been carried out in EpiSCs. We used reprogramming intermediates generated from neural stem cells (NSCs) through retroviral expression of *Oct4*, *Klf4* and *cMyc*, to address whether *Zmym2* might also inhibit *Nanog*-induced reprogramming in a somatic cell context. These cells were stably transfected with a PiggyBac (PB) *Nanog* transgene and subjected to transient transfection with either control or *Zmym2* siRNA. In keeping with the results obtained in EpiSCs, *Zmym2*KD increased somatic cell reprogramming more than two-fold ( Figure 2A). To ascertain whether *Zmym2*KD could reprogram somatic cells in the absence of *Nanog*, the same experiment was carried out in *Nanog* ^-/-^ somatic cells ^10^. These were stably transfected with a rescue *Nanog* transgene or a corresponding EV transgene, and reprogrammed after control KD or *Zmym2*KD. As seen in Figure 2B, *Zmym2*KD also enhanced Nanog-mediated reprogramming in neural stem cell derived reprogramming intermediates. *Zmym2*KD was not sufficient to overcome the requirement for *Nanog* in somatic cell reprogramming, though we confirmed that it enhances *Nanog*-mediated reprogramming in this context.

As *Zmym2*KD increases *Nanog*-mediated reprogramming efficiency, we decided to carry out the converse experiment and investigate whether *Zmym2* overexpression could impair reprogramming. Four lines of EpiSCs were generated which stably overexpressed either *Nanog*, *Zmym2,* or both ( Figure 2C), and these were induced to reprogram by transfer to 2i Plus LIF medium. As expected, *Nanog* overexpression resulted in efficient EpiSC reprogramming while *Zmym2* overexpression alone had no reprogramming activity ( Figure 2D). However, when *Zmym2* overexpression was combined with *Nanog* overexpression, it reduced reprogramming efficiency 8-fold relative to *Nanog* alone ( Figure 2D). To test this result in an independent cell system, mouse embryonic fibroblast (MEF)-derived reprogramming intermediates expressing retroviral *Oct4*, *Klf4*, *cMyc* and *Sox2* and a *Nanog* transgene were transfected with either Empty Vector (EV) or a *Zmym2* expression cassette ( Figure 2E). *Nanog* alone led to highly efficient complete reprogramming ( Figure 2F, G, H) whereas the addition of *Zmym2* completely prevented reprogramming.

**Figure 3.**
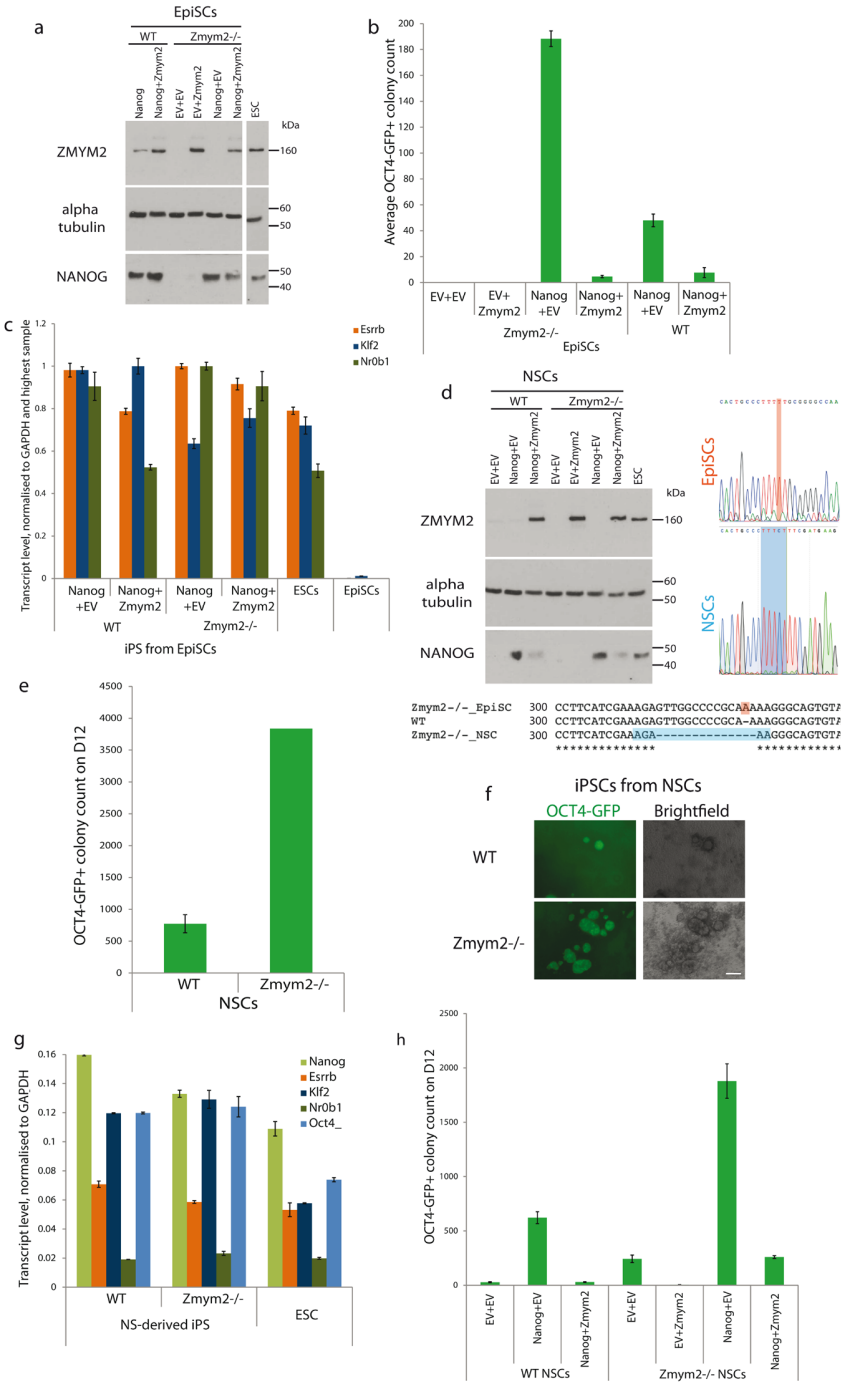
*Zmym2* knockout enhances *Nanog*-mediated reprogramming in epiblast stem cells (EpiSCs) and somatic cells. **a**,Western blot analysis of starting populations of EpiSCs. Both alleles of *Zmym2* were disrupted with the CRISPR/Cas9 system. *Nanog*, *Zmym2* or both were stably overexpressed. **b**, Average GFP ^+ ^colony count of reprogrammed EpiSCs on D12 for *Zmym2* ^-/-^ and WT EpiSCs, per 50,000 cells plated, three replicates. **c**, Gene expression analysis of the resulting iPSCs shows them to be faithfully reprogrammed, in contrast to the starting population of EpiSCs. **d**, Western blot analysis of starting populations of NSCs. Both alleles of *Zmym2* were disrupted with the CRISPR/Cas9 system. *Nanog*, *Zmym2* or both were stably overexpressed. Clustal Omega multiple sequence alignment of KO clones with the WT sequence and reverse complement sequencing traces of *Zmym2*KO EpiSCs and NSCs. **e**, Average iPSC colony count after reprogramming of *Zmym2* ^-/-^ and WT NSCs per 75,000 cells plated. **f**, Fluorescence and brightfield images of iPSCs generated from *Zmym2* ^-/-^ and WT NSCs after retroviral *Oct4, Klf4 and cMyc* overexpression and exposure to 2i Plus LIF. Scale bar 500μm. **g**, Gene expression analysis of the resulting WT and *Zmym2* ^-/-^ iPSCs by qPCR. **h**, NSCs were stably transfected with *Nanog*, *Zmym2* or both and reprogrammed with retroviral *Oct4, Klf4 and cMyc* in 2i Plus LIF. Average colony count for *Zmym2* ^-/- ^and WT NSCs per 75,000 cells plated, three replicates. Reprogramming counts are shown as mean +/- SEM. qPCR quantifications are shown as mean of three technical replicates +/- SD, normalised to *Gapdh* transcript levels.

In order to investigate the effect of *Zmym2* loss in reprogramming, *Zmym2* ^-/-^ EpiSCs were generated by CRISPR/Cas9-mediated mutagenesis ( Figure 3A). WT and *Zmym2* ^-/-^ EpiSCs were then stably transfected with *Nanog* or *Zmym2* or both ( Figure 3A) and allowed to reprogram. Similar to previous results, *Zmym2* overexpression decreased *Nanog*-mediated reprogramming in wild type cells ( Figure 3B). In agreement with KD experiments, *Zmym2* knockout increased *Nanog*-induced reprogramming by about 4-fold ( Figure 3B). This effect was rescued by the addition of transgenic *Zmym2* ( Figure 3B). WT and *Zmym2* ^-/-^ iPSCs were indistinguishable by gene expression analysis of pluripotency-associated markers ( Figure 3C).

To verify this result in an independent cell system, WT or *Zmym2 ^-/-^* NSCs were also generated by CRISPR/Cas9-mediated mutagenesis ( Figure 3D). They were then retrovirally transduced with *Oct4, Klf4* and *cMyc* and allowed to reprogram. As in EpiSCs, *Zmym2* ^-/-^ NSCs reprogrammed with much higher efficiency than their WT counterparts ( Figure 3E, F). Both WT and *Zmym2* ^-/-^ iPSCs had gene expression profiles similar to those of control ESCs, demonstrating complete reprogramming ( Figure 3G). Both WT and *Zmym2 ^-/-^* NSC lines were then stably transfected with *Nanog*, *Zmym2,* or both, to create a rescue system for reprogramming ( Figure 3D). Again, *Zmym2* knockout increased *Nanog*-induced reprogramming by 3-fold ( Figure 3H), whereas its overexpression eliminated the enhancement of reprogramming by *Nanog* ( Figure 3H).

### *Zmym2* reduces ESC self-renewal

*Zfp281* is known to enable *Nanog* autorepression ^24^ so we tested whether *Zmym2* levels had any effect on *Nanog* transcript levels. Neither KD nor overexpression lines had any change in *Nanog* transcript or protein levels ( Figure 1B, 1C, Figure 2C, Figure 3C, 3G), suggesting that *Zmym2* does not act through the regulation of *Nanog* expression.

**Figure 4.**
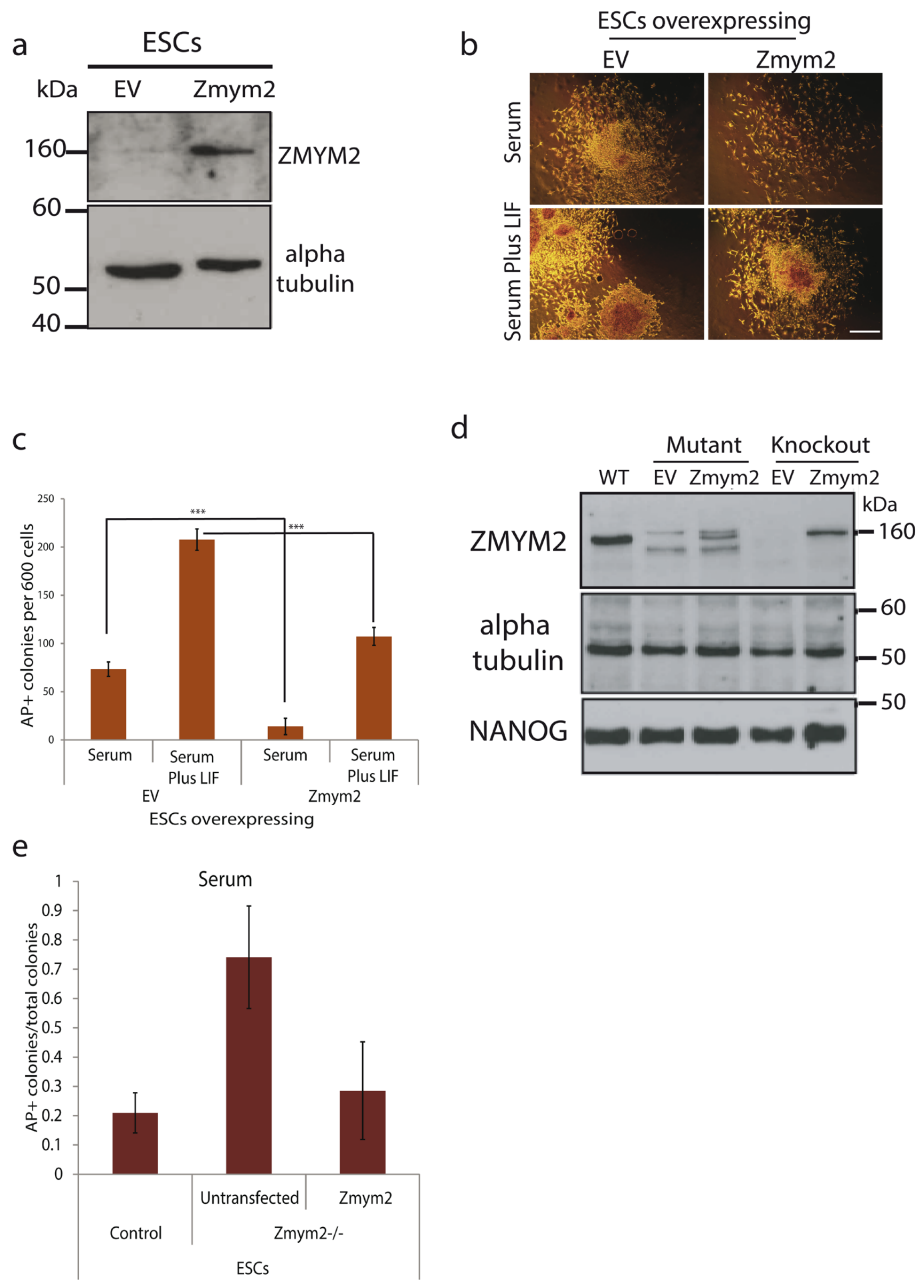
*Zmym2* inhibits embryonic stem cell (ESC) self-renewal. **a**, Western blot analysis of ESCs stably overexpressing *Zmym2* or a corresponding EV transgene. **b**, Brightfield images of alkaline phosphatase stained plates of EV- of *Zmym2*-overexpressing ESCs after 6 days in medium containing either serum or Serum+LIF. Scale bar 500μm. **c**, Scores of undifferentiated colonies on D6 after plating in Serum or Serum Plus LIF. Mean of 3 replicates +/-SEM. **d**, *Zmym2* was disrupted using CRISPR/Cas9. Clones were stably transfected with *Zmym2* or a corresponding EV transgene. Western blot of two resulting ESC clones; the knockout clone was sequenced and used for further experiments. **e**, Scores of undifferentiated colonies on D6 after plating WT, *Zmym2* ^-/-^ and *Zmym2* rescued lines in Serum or Serum Plus LIF indicate that *Zmym2* deletion may impede differentiation in the absence of LIF. Mean of 3 replicates +/- SEM.

*Nanog* was first discovered for its role in the self-renewal of ESCs ^8, 9^. As ZMYM2 is a NANOG interactor, we hypothesised that it might also inhibit *Nanog*’s self-renewal-promoting capacity. In order to address this, *Zmym2* was stably transfected into ESCs. These cells were plated alongside EV controls ( Figure 4A) in Serum-containing medium with LIF for 6 days, to maintain pluripotency in some cells while allowing others to differentiate. Alkaline-phosphatase staining was carried out and colonies were scored. *Zmym2*-overexpressing ESCs exhibited greater spontaneous differentiation than control ESCs ( Figure 4B, C).

In order to investigate whether *Zmym2*KO impedes ESC differentiation, both *Zmym2* alleles were knocked out using the CRISPR/Cas9 system as previously described. ZMYM2 ^-/- ^cells and FLAG-tagged *Zmym2* rescue cells were generated ( Figure 4D). These lines were plated alongside the parental WT ESC line in the absence of LIF for 6 days, alkaline phosphatase stained and colonies were scored by morphology. *Zmym2*KO increased the proportion of undifferentiated colonies ( Figure 4E). This was rescued by transgenic *Zmym2* expression. This is in agreement with a recently published Cas9 ESC differentiation screen which demonstrated that *Zmym2*KO ESCs resist differentiation ^25^.

**Figure 5.**
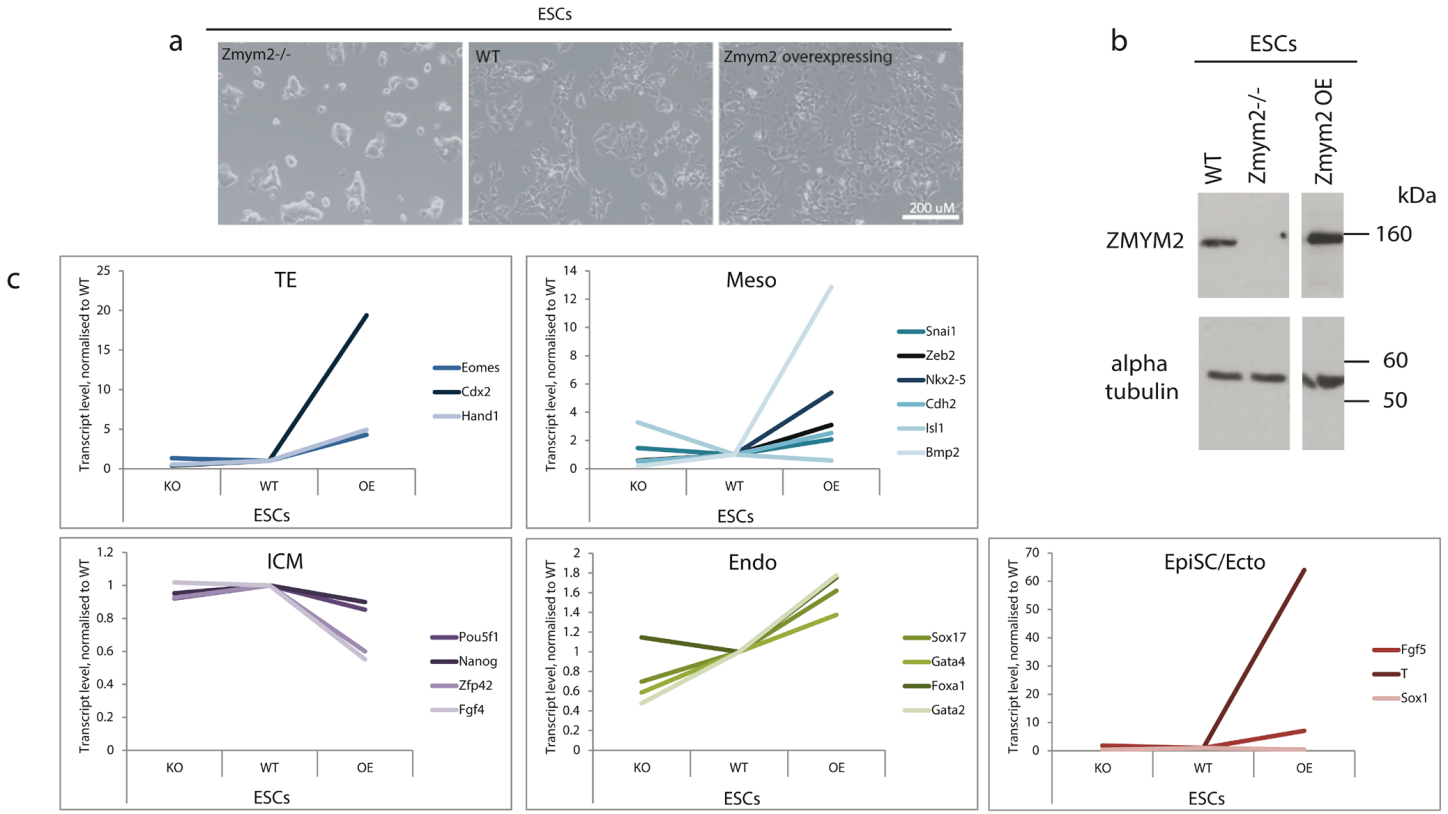
*Zmym2* overexpression correlates with the mis-expression of early lineage markers. **a**,Brightfield images of Cas9-generated *Zmym2* ^-/-^ ( Figure 4D), WT and *Zmym2* overexpressing embryonic stem cells (ESC) **** in Serum Plus LIF-containing medium. **b**, Western blot analysis of these ESC lines. **c**, RNA Seq analysis was performed on the lines shows an upregulation in the transcript levels of early lineage specifiers of trophectoderm (TE), ectoderm and EpiSCs (EpiSCs/Ecto), mesoderm (meso) and endoderm (endo) and a corresponding decrease in the transcript levels of inner cell mass (ICM) markers in *Zmym2* overexpressing ESCs. Conversely, *Zmym2* ^-/-^ ESCs have lower transcript levels of early lineage markers.

To address the global effects of *Zmym2* loss on the transcriptome, mRNA from *Zmym2* KO, WT and overexpressing ESCs ( Figure 5A, B) were subjected to mRNA-Seq after culture in Serum Plus LIF-containing medium. *Zmym2* overexpressing cells had higher transcript levels of many lineage specifiers than control cells including early ectodermal, mesodermal and endodermal markers, as well as trophectodermal markers, after normalisation to housekeeping genes. In addition, they had reduced transcript levels of a number of pluripotency-associated genes. Conversely, *Zmym2*KO cells had reduced expression of differentiation markers. In conclusion, *Zmym2* inhibits reprogramming and promotes differentiation. It has a global effect on the transcriptome of ESCs, increasing the transcription of differentiation-associated genes and reducing pluripotency-associated transcripts. 

## Discussion

In this work, we show that *Zmym2* represents a significant barrier to *Nanog*-mediated reprogramming. We observed consistent results using gain and loss of function assays in many different reprogramming systems, including EpiSCs, fibroblasts and neural stem cells. This corroborates results obtained by other groups working on RNAi in human cell reprogramming ^26^. Therefore, ZMYM2 may play a similar role in the control of NANOG in mouse and human.

We also observe that *Zmym2* promotes embryonic stem cell differentiation. This has also been reported in a Cas9 screen for differentiation-promoting factors ^25^. Future work could examine whether the absence of *Zmym2* impacts mouse development and elucidate its role *in vivo*.

In conclusion, this work has elucidated the key role of *Zmym2* as a barrier to reprogramming and a differentiation-promoting transcription factor. This is particularly interesting as many previous studies of *Nanog*’s mechanism of action have identified positive regulators of its activity ^11, 13^. We have shown both more effective reprogramming and less differentiation upon removal of *Zmym2* demonstrating how the tight control of NANOG by its binding partners exerts a directive influence on cell identity transitions, both entering and exiting the pluripotent state. 

## Data availability

### Underlying data

RNASeq data from WT, Zmym2 knockout- and Zmym2 overexpressing- E14tg2a mouse embryonic stem cells, Accession number GSE130317: http://identifiers.org/geo:GSE130317

Open Science Framework: ZMYM2. https://doi.org/10.17605/OSF.IO/TFKHR ^27^

This project contains the following underlying data:

- 1c.pdf (x-ray films for Western blot in Figure 1c)
- 1g.jpg (x-ray films for Western blot in Figure 1g)
- 2h.jpg (alkaline phosphatase staining for colony counts in Figure 2h)
- 3a d 5b.pdf (x-ray films for Western blot in Figure 3a, 3d and Figure 5b)
- 3f_1.tif (Image taken at 488nm of Oct4-GFP ^+^ colonies for Figure 3f)
- 3f_2.tif (Brightfield image of Oct4-GFP ^+^ colonies shown in 3f-1)
- 3f_3.tif (Image taken at 488nm of Oct4-GFP ^+^ colonies for Figure 3f)
- 3f_4.tif (Brightfield image of Oct4-GFP ^+^ colonies shown in 3f-3)
- 4a.pdf (x-ray films for Western blot in Figure 4a)
- 4b_1.tif (Brightfield image of alkaline phosphatase-stained well shown in 4b)
- 4b_2.tif (Brightfield image of alkaline phosphatase-stained well shown in 4b)
- 4b_3.tif (Brightfield image of alkaline phosphatase-stained well shown in 4b)
- 4b_4.tif (Brightfield image of alkaline phosphatase-stained well shown in 4b)
- 4c_1.jpg (alkaline phosphatase staining for colony counts in Figure 4c)
- 4c_2.jpg (Coomassie staining for colony counts in Figure 4c)
- 4c_3.jpg (alkaline phosphatase staining for colony counts in Figure 4c)
- 4c_4.jpg (Coomassie staining for colony counts in Figure 4c)
- 4d_1.pdf (x-ray films for Western blot in Figure 4d)
- 5a KO ESCs.jpg (Brightfield image of KO ESCs shown in 5a)
- 5a WT ESCs.jpg (Brightfield image of WT ESCs shown in 5a)
- 5a Zfp198BSD E14.jpg (Brightfield image of OE ESCs shown in 5a)
- All data Zmym2 Lawrence.xlsx (All GFP ^+^ colony counts, Coomassie colony counts, alkaline phosphatase ^+^ colony counts, and qPCR Ct values underlying this paper)
- CC0: Results.pdf (PDF confirming that these results have been declared CC0)

Data are available under the terms of the Creative Commons Zero "No rights reserved" data waiver (CC0 1.0 Public domain dedication).
