# Supplementary material for: ZMYM2 inhibits NANOG-mediated reprogramming
Source: Wellcome Open Res. 2019 Jun 6;4:88. [Version 1] doi: 10.12688/wellcomeopenres.15250.1 (PMC6640293; doi:10.12688/wellcomeopenres.15250.1)
Supplement: Peer review history

# Peer review: Reviewer response for version 1

**DOI:** 10.21956/wellcomeopenres.16645.r35874

**Published:** 2019-08-02

**Authors:** Junjun Ding

**Affiliations:** 1 Zhongshan School of Medicine, Sun Yat-Sen University, Guangzhou, China

This manuscript proves that *Zmym2 *acts as a repressor in *Nanog*-mediated reprogramming through gain and loss of function assays. The novelty of this manuscript is moderate, and there are some problems that needs to be improved.

**Main points:**

1. The information in the introduction is too simple and incomplete. There are lots of proteins in NANOG interactome, please give more detailed reasons why the author chose the ZMYM2 as the research object. Also, the nature and function of ZMYM2 that has been studied so far could be introduced.
2. In the section of “Zmym2 reduces ESC self-renewal”, the author said that “Neither KD nor overexpression lines had any change in Nanog transcript or protein levels”, however, as is shown in Figure 2e, when Zmym2 is overexpressed, the transcript level of Nanog also increases, which seems that the expression of Nanog is regulated by Zmym2.
3. In the discussion part, could you please give some possible mechanism about why Zmym2 have a pronounced positive effect on Nanog-mediated reprogramming but a minimal effect on reprogramming of no-Nanog-mediated?
4. It seems that the authors were not serious enough when making figures. The detail of this criticism would be listed in the “minor points” section.

**Minor points:**

1. It’s a bit confusing to emphasize that NANOG is a “homeodomain-containing” transcription factor in both the abstract and the first paragraph of the introduction.
2. The method of reprogramming epiblast stem cell is missing.
3. Font size is expected to be 8-10 pt in all figures. For example, the fonts are too large in Figure 4d while the ones are too small in Figure 3.
4. The upper half part of error bar of the “ESCs” is missing in Figure 1d.
5. The meaning of “Pool” should be explained in the figure legend of Figure 1.
6. What dose “Oct4_” mean in Figure 2c and 3g?
7. It’s inaccurate to regard neural stem cells as somatic cells in the title of Figure 3.
8. “Zmym2” is expected to be italic in all figures.
9. In the result of western blot, the “alpha tublin” should be written as “α-TUBLIN”.
10. The P value should be provided in every bar graph.

I confirm that I have read this submission and believe that I have an appropriate level of expertise to confirm that it is of an acceptable scientific standard, however I have significant reservations, as outlined above.

# Peer review: Reviewer response for version 1

**DOI:** 10.21956/wellcomeopenres.16645.r35873

**Published:** 2019-07-17

**Authors:** Tom Burdon

**Affiliations:** 1 The Roslin Institute and R(D)VS, University of Edinburgh, Edinburgh, UK

This thorough study investigates a role for Zmym2 in Nanog-mediated reprogramming, and in ESC differentiation. By using RNA knock-down, gene knock-out and over-expression in a series of bespoke reprogramming tests, and a standard ESC self-renewal assay, the authors have comprehensively demonstrated that Zmym2 antagonises Nanog-driven reprogramming and promotes the loss of pluripotency in ESCs. This novel finding significantly extends the previously reported observation that Zmym2 physically interacts closely with the Nanog protein in ESCs. 

 Nanog is a member of a group of ancillary pluripotency-associated factors that are not essential in all circumstances, and may vary in their importance in embryo stem cells of different species. Given the significant protein sequence divergence of Nanog outside the core DNA binding domain, future studies may investigate what regions of Nanog (or partners) interact with Zmym2, and the degree to which this interaction is conserved in Nanog-expressing cell types, and between species.

 However, questions that might be answered using the available data and included directly in this present report, are:  

1. How does Zmym2 expression affect Nanog target gene expression? Analysis of the RNA seq data for effects on Nanog target expression in the KO, WT and OE cells might shed light on how Zmym2 influences Nanog function.
2. Similarly, does Zmym2 affect self-renewal/differentiation signalling in ESCs? The relevant data presented in Figure 5 shows striking induction of Cdx2 and Brachyury as well as BMP2. Since Cdx2 and Brachyury are known targets for Wnt/b-catenin signalling, is there evidence of Zmym2 upregulation of this pathway. Or is their induction mediated via expression of BMP2, and accompanied by upregulation of other BMP signalling/targets?

Other points for consideration are:

- The introduction/discussion would benefit from mentioning background information on Zmym2, as well as its pattern of expression during early differentiation (upregulation during EpiSC formation?). Could there be any significance to Zmym2/FGFR association and Nanog function?
- Clarification in the first part of the results that the "control" ZFP281 is a Nanog interacting protein.
- Minor typo - Figure legend 2 “ 75,000 plates NSCs” - “plated”.

I confirm that I have read this submission and believe that I have an appropriate level of expertise to confirm that it is of an acceptable scientific standard.

# Peer review: Reviewer response for version 1

**DOI:** 10.21956/wellcomeopenres.16645.r35716

**Published:** 2019-06-21

**Authors:** Andrew Johnson

**Affiliations:** 1 Genetics, University of Nottingham, Nottingham, Nottingham, NG7 2UH, UK

I think this is a very good and interesting paper that addresses an important issue in stem cell biology. Some years ago, NANOG was identified as a “gateway to pluripotency”, so how its activity is regulated during reprogramming is of considerable interest. Here the authors investigate the activity of a transcription factor called ZMYM2 that they have identified as a component of the NANOG interactome, and they show that it inhibits NANOG activity both during reprogramming and self-renewal. Other studies, including work by the same lab, have demonstrated the augmentation of NANOG activity by components of the interactome; this is the first to my knowledge to show an inhibitory activity. Interestingly, ZMYM2 does not inhibit NANOG expression, so its effects must be biochemical, and future studies to elucidate these biochemical activities will also be of considerable interest in the field. The studies are thorough, well executed and convincing. For example, the authors use several different reprogramming contexts, including epiSC, fibroblasts and neural stem cells to demonstrate the inhibitory effects of ZMYM2 on reprogramming. They also use a combination of knockout and overexpression experiments, which clearly demonstrate complementary results that reinforce the paper’s conclusions. Their data also show that ZMYM2 inhibits self-renewal by promoting differentiation, suggesting it may play an interesting role in cell-specification *in vivo*. In all this paper is certainly worthy of indexing and it will be of interest to the field. I think a couple of minor issues should be addressed.

 First, the second line of the Introduction states that pluripotent cells possess the ability to self-renew. However, this is only true of pluripotent stem cells maintained in culture. The pluripotent cells in an embryo do not necessarily self-renew, particularly in non-mammalian systems where the number of pluripotent cells increases by cleavage rather than expansion of an epiblast.

 In Figure 4d the difference between the mutant and a knockout ESC lines should be made clearer. The different patterns of ZMYM2 in the two lines should also be explained in the text.

 Figure 4e does not show ESC in serum plus LIF.

I confirm that I have read this submission and believe that I have an appropriate level of expertise to confirm that it is of an acceptable scientific standard.
